# Factors associated with quality of life in Italian children and adolescents with IBD

**DOI:** 10.1038/s41598-021-97661-1

**Published:** 2021-09-10

**Authors:** Simona Gatti, Giada Del Baldo, Giulia Catassi, Andrea Faragalli, Marina Aloi, Matteo Bramuzzo, Giulia D’Arcangelo, Enrico Felici, Maurizio Fuoti, Sara Lega, Roberto Panceri, Maria Pastore, Francesca Penagini, Rosaria Gesuita, Carlo Catassi

**Affiliations:** 1 Department of Pediatrics, Polytechnic University of Marche, G. Salesi Children’s Hospital, Via Corridoni 11, 60123 Ancona, Italy; 2 Department of Maternal and Child Health, Pediatric Gastroenterology and Liver Unit, Umberto I Hospital, Sapienza University of Rome, Viale del Policlinico 105, 00161 Rome, Italy; 3 Centre of Epidemiology and Biostatistics, Università Politecnica delle Marche, Via Tronto 10/a, 60126 Ancona, Italy; 4 Digestive Endoscopy and Nutrition Unit, Institute of Child and Maternal Health, IRCCS “Burlo Garofolo”, Via dell’Istria 65, 34137 Trieste, Italy; 5 Pediatrics and Pediatric Emergency Unit “U.Bosio” Center for Pediatric Digestive Diseases, The Children Hospital, Azienda Ospedaliera SS. Antonio e Biagio e Cesare Arrigo, Spalto Marengo 46, 15121 Alessandria, Italy; 6 Gastroenterology and GI Endoscopy, University Department of Pediatrics, Children’s Hospital, Piazzale Spedali Civili 1, 25123 Brescia, Italy; 7 Clinica Pediatrica, Università Milano Bicocca, Fondazione MBBM, Ospedale San Gerardo, Via Cadore, 20900 Monza, Italy; 8 IRCCS Casa Sollievo della Sofferenza-Pediatria, Viale Padre Pio, 7d, 71013 San Giovanni Rotondo, Italy; 9 Clinica Pediatrica, Ospedale dei Bambini “V. Buzzi”, Università degli Studi di Milano, Via Lodovico Castelvetro 32, 20154 Milano, Italy

**Keywords:** Gastroenterology, Health care

## Abstract

Improving the quality of life (QoL) is crucial in the management of pediatric inflammatory bowel disease (IBD). We aimed to (1) Validate the IMPACT-III questionnaire in Italian IBD children; (2) explore factors associated to QoL in pediatric IBD. Internal consistency, concurrent validity, discriminant validity and reproducibility of the Italian version of the IMPACT-III questionnaire was measured in IBD children/adolescents in 8 centers. Associations between patient and disease characteristics and the IMPACT-III domains were analyzed through quantile regression analysis. The IMPACT-III questionnaire, collected in 282 children with IBD (median age: 14.8 years; IQR 12.4–16.4) showed a median total score of 76 (IQR 67–83). Female gender, active disease and age were negatively associated with the total IMPACT-III score. Specifically, female gender was negatively associated with the Bowel/Systemic Symptoms, Emotional and Treatment domain scores, while disease activity was significantly associated with Bowel Symptoms and Treatment/Interventions reported QoL. The IMPACT- III showed good internal consistency (Cronbach’s alpha coefficient = 0.87, 95% CI 0.85–0.89) and reproducibility (Concordance Correlation Coefficient = 0.66, 95% CI 0.57–0.74). In Italian children with IBD active disease, female gender and adolescence are associated to a worse QoL, indicating the need of more attention in this subgroup of young patients. IMPACT-III questionnaire is a reliable instrument to measure QoL in Italian children.

## Introduction

Inflammatory bowel diseases (IBD) including Crohn’s disease (CD), Ulcerative Colitis (UC) and IBD unclassified (IBD-U), are chronic inflammatory conditions characterized by an unpredictable clinical course, alternating periods of remission and clinical relapses. About 25% of IBD cases are diagnosed in childhood (< 18 years) and the global incidence of pediatric IBD is increasing worldwide^1^. Children with IBD face a lifelong disabling condition, experiencing an increased risk of psychological distress and social disruption, and report impaired quality of life (QoL) in comparison to healthy controls^2^.

Improvement of patient-reported outcomes (PROs), such as QoL, has recently been recognized as an important target of IBD management^3–5^, therefore a robust, culturally adapted and reliable instrument is necessary to accurately measure QoL, both in clinical and research settings. The IMPACT III questionnaire is the most frequently used scale to assess disease specific QoL in children with IBD^6,7^. The original version has been translated in several languages but so far, a rigorous cultural validation has been conducted only in few European countries (UK, Switzerland and Croatia^8–10^).

Several factors, including individual and disease specific characteristics, have been reported to impact QoL in IBD. It has been described that disease activity is the main predictive factor of IBD-related QoL in adults^2^, however pediatric data are lacking. An accurate knowledge of the demographic and clinical variables affecting QoL in a specific geographical context can be useful in the interpretation of reported QoL, with the ultimate goal of improving care of pediatric patients with IBD.

The aim of the present study was to assess the validity of the IMPACT III questionnaire as a measure of QoL in Italian IBD children. Furthermore, we aimed to identify patient and disease characteristics associated with QoL of children with IBD.

## Methods

### Patients

Children and adolescents (age 8–18 years) with a confirmed diagnosis of IBD (according to the revised Porto criteria)^11^, established at least 6 months prior to enrolment, were invited to participate. Patients with cognitive impairment, severe co-morbidities and presence of an ostomy were not included, as their IBD related QoL was likely to be significantly influenced by factors different from the majority of IBD children who do not present these complications. Children were enrolled at 8 regional referral pediatric IBD centers, equally distributed across the Italian territory (Ancona, Brescia, Milano, Pesaro, Roma, San Giovanni Rotondo, Trieste and Monza), both in out-patient and in-patient setting, irrespective of disease activity. Informed consent was obtained by the parents or guardians. The study was approved by the coordinating center Ethics Committee (“Comitato Etico Regione Marche, CERM”, protocol number 2016–0359 OR).

At the time of enrolment, the following data were collected: age at diagnosis, diagnosis, location and phenotype according to Paris classification^11^, recent relapses (12 months), previous surgery, fecal calprotectin levels and growth. Body weight (BW) and height (H) were collected and body mass index (BMI) was calculated as weight/height^2^ (kg/m^2^). BW, H, and BMI z-scores were then calculated. Disease activity was measured using the weighted Pediatric Crohn’s Disease activity index (wPCDAI)^12^ or Pediatric Ulcerative Colitis Index (PUCAI)^13^ for CD and UC patients respectively plus Physician Global assessment (PGA). Physicians indicated whether patients had quiescent, mild, moderate, or severe PGA, according to the definitions of the ImproveCareNow network [https://improvecarenow.org]^14^. Fecal calprotectin level > 250 µg/g was considered as an indicator of active mucosal disease.

### Quality of life questionnaires

Generic health related quality of life (HRQoL) was assessed using the generic HRQoL questionnaire for children, Pediatric Quality of Life Inventory (PedsQL, Italian version) designed by J. Varni^15^. PedsQL is a 24-items questionnaire with proven validity and reliability in pediatric patients, exploring 4 different domains (Physical, Emotional, Social and School Functioning). The Total score, the Physical Health Summary score and Psycho-Social Health summary score were calculated, according to the author guidelines.

Disease specific QoL was assessed using the Italian version of the IMPACT-III questionnaire. The original English version of the IMPACT-III was forward- and backward-translated and culturally adapted according to the guidelines outlined by Beaton and colleagues^16^ in 2008 by other authors (data not published), therefore an Italian version certified by the developer (Anthony Otley) was already available and obtained with permission*.* The questionnaire contains 35 IBD specific items, exploring six different domains: Bowel Symptoms, Emotional functioning, Social functioning, Systemic symptoms, Body image and Treatment/Intervention. The responses for each question are on a five-point Likert scale (scores are 0–4, with 0 indicating the highest HRQoL and 4 indicating the lowest). As per author’s guidelines scores are then linearly and reversely transformed to a range of 0–100, with 0 indicating the lowest HRQoL and 100 indicating the highest HRQoL. Missing data on IMPACT-III scores were treated as indicated in the IMPACT-III User’s Guide, total and domain scores were computed as the mean of all completed items^17^.

Children were requested to fill in both PedsQL and IMPACT III without parental help, but could ask the administering physician for information.

To assess the test–retest reliability children were contacted by phone after 4–8 weeks. If the patient reported no change in clinical symptoms and no further treatments had been introduced since the enrolment, children were asked to compile a second copy of the IMPACT III and return it to the refering center.

### Statistical analysis

#### Validation of the Italian version of the IMPACT III questionnaire: reliability and validity evaluation

The questionnaire’s validation was carried out using sample data. Internal reliability was assessed using Cronbach’s *α* coefficient, analyzing the consistency of each domain. The distributions of each domain score were asymmetric, hence a non-parametric approach was chosen. In order to evaluate the concurrent validity, the correlation between PedsQL and IMPACT III total score was analyzed. A correlation analysis was performed between each domain of IMPACT III and PedsQL’s Psychosocial Health Summary Score, Physical Health Summary Score by Spearman’s correlation coefficient and 95% Confidence Intervals (95% CI).

Discriminant validity was analyzed to determine whether the questionnaire (total score and domain scores) was able to discriminate patients in remission from patients with moderate/severe activity, according to the wPCDAI and PUCAI results. The Wilcoxon sum-rank test was used to evaluate differences between domain scores of the two groups.

Data collected from patients with a stable disease (no change in reported symptoms and no additional treatments), who had completed IMPACT-III questionnaire twice, were analyzed for testing the reproducibility, estimating the median of the differences in the domains of IMPACT III score and 95% Confidence Intervals (95% CI) and by means of Concordance Correlation Coefficient (CCC)^18^ and 95% Confidence Intervals (95% CI); CCC quantifies the agreement between two measurements of the same variable.

#### IMPACT-III questionnaire analysis

A descriptive analysis of the main characteristics of subjects was performed to compare CD and UC patients. Medians and interquartile ranges (IQR), absolute and percentage frequencies were used to summarize quantitative and qualitative variables respectively. Wilcoxon rank sum test or Chi-square/Fisher exact test were performed to evaluate differences between groups.

The distributions of scores of each IMPACT-III domain according to participating centres were summarized using medians and IQRs and comparisons between centres were performed using Kruskal–Wallis test.

Multiple quantile regression analysis was used to evaluate the impact of subject characteristics and type of disease on the median value of each IMPACT-III domain distribution (dependent variable). The following independent variables were considered in each model: gender, age, disease duration, BMI Z-score, disease severity measured by PGA and PUCAI/wPCDAI (both dichotomized as remission and active disease), type of disease (CD versus UC), occurrence of relapses during the last year and surgery. Quantile regression is a non parametric method that does not make any model assumption that may not hold and provides a more complete view of the effect of each variable on the global distribution of IMPACT-III scores. Nine deciles of the distributions of scores were evaluated when the total score was analyzed. Results for IMPACT-III total score domain were showed in a plot where the x-axis indicated the 9 deciles of the response distribution (score) and y-axis exhibited the effect of each variable (regression coefficients) and 95% confidence bands (grey area). If the confidence interval included 0, the estimates could not be considered statistically different from 0.

### Ethical approval

All procedures performed in this study were in accordance with the ethical standards of the institutional and/or national research committee and with the 1964 Helsinki declaration and its later amendments or comparable ethical standards. The study was approved by the coordinating center Ethics Committee (“Comitato Etico Regione Marche, CERM”, protocol number 2016–0359 OR). Informed consent was obtained from all individual participants included in the study.

## Results

A total of 282 children with IBD (median age 14.8 years, IQR 12.4–16.4) were consecutively enrolled over 19 months, after informed consent was obtained. One-hundred and fifty-five children had CD (54.9%), 123 had UC (43.6%) and 4 had IBD-U (1.4%). At the time of enrolment, the majority of subjects was in clinical remission (68.7%; 95% CI 61.1–75.2), with only 5 patients (3 with UC) having severe disease. *Table **1* shows the clinical characteristics of enrolled subjects. No significant difference between CD and UC subjects was found, except a higher prevalence of males in CD subjects (p = 0.010) and a higher number of relapses during the last year in the UC group (p = 0.012). The IMPACT-III questionnaire was completely filled in by 272 children (97.8% of the enrolled population), with 6 children missing only one response.

**Table 1:** Demographic and clinical characteristics of subjects according to type of disease.

	CD (n = 155)	UC (n = 123)	p
Gender [M, n (%)]	97 (62.6%)	57 (46.3%)	0.010^a^
Age [years, median (IQR)])	15.2 (12.6; 16.7)	14.6 (12.5; 16.5)	0.258^b^
Disease duration [years, median (IQR)]	2.9 (1.4; 5.2)	3.8 (1.7; 6.2)	0.052^b^
Z-score weight [median (IQR)]	− 0.5 (− 1.2; 0.3)	− 0.4 (− 0.9; 0.2)	0.663^b^
Z-score BMI [median (IQR)]	− 0.48 (− 1.1; 0.2)	− 0.4 (− 1.19; 0.2)	0.795^b^
**Disease activity**			
N. of relapses [median (IQR)]	0 (0; 3)	0 (0; 4)	0.012^b^
**PUCAI/w-PCDAI [n (%)]**			0.492^c^
Remission	111 (73.5%)	90 (75%)	
Mild	33 (21.9%)	20 (16.7%)	
Moderate	5 (3.3%)	7 (5.8%)	
Severe	2 (1.3%)	3 (2.5%)	
**PGA [n (%)]**			0.138^c^
Remission	106 (70.2%)	87 (75.5%)	
Mild	37 (24.5%)	17 (14.8%)	
Moderate	7 (4.6%)	9 (7.8%)	
Severe	1 (0.7%)		
Surgery [yes, n (%)]	9 (11.25%)	6 (8.45%)	0.439^a^
Fecal calprotectin > 250 ug/g [yes, n (%)]	32 (36.4%)	30 (44.1%)	0.800^a^
Fecal calprotectin [ug/g; median (IQR)]	118 (37; 427)	193 (32; 707)	0.364^b^

p-values refer to: ^a^Chi-square test; ^b^Wilcoxon test; ^c^Fisher Exact test.

*IQR* interquartile range.

The number of patients and IMPACT-III domain scores for each participating centre is shown in Supplementary Table S1. On average, 35 subjects were recruited in each centre, with two centres (Pesaro, Monza) with the lowest numbers of participants (6 and 10 respectively) and Trieste with the highest number of participants. There were no significant differences in the total score and in the subdomains of the questionnaire between participating centres, except for a significant difference in the Bowel Symptoms domain score between San Giovanni Rotondo and Trieste.

### IMPACT-III validation

#### Internal consistency and concurrent validity

Internal consistency and concurrent validity results are shown in Tables 2 and 3, respectively. The overall Cronbach's alpha coefficient showed a very high consistency among all the items of the questionnaire (α = 0.87, 95% CI 0.85–0.89). Cronbach's alpha coefficients showed high internal consistency for the Emotional Functioning and Systemic Symptoms domains; a good internal consistency for Bowel Symptoms, Social Functioning and Body Image domains. Cronbach’s alpha of the Treatment Intervention domain was 0.52, showing a fair internal consistency.

**Table 2:** Internal consistency.

IMPACT III domains	Cronbach's alpha	
	α	95% CI
Bowel symptoms	0.73	(0.69; 0.78)
Emotional functioning	0.83	(0.8; 0.86)
Social functioning	0.72	(0.67; 0.77)
Systemic simptoms	0.8	(0.76; 0.84)
Body imgae	0.65	(0.58; 0.72)
Treatment Interventions	0.52	(0.42; 0.62)

*95% CI* 95% Confidence Interval.

**Table 3:** Concurrent validity.

IMPACT III domains	PEDSQL domains			
	Psychosocial health summary score		Physical health summary score	
	r (95%CI)	p	r (95% CI)	p
Bowel symptoms	0.52 (0.43; 0.61)	< 0.001	0.64 (0.56; 0.70)	< 0.001
Emotional functioning	0.61 (0.53; 0.68)	< 0.001	0.54 (0.45; 0.62)	< 0.001
Social functioning	0.53 (0.44; 0.61)	< 0.001	0.44 (0.34; 0.53)	< 0.001
Systemic simptoms	0.63 (0.55; 0.70)	< 0.001	0.67 (0.60; 0.73)	< 0.001
Body imgae	0.42 (0.32; 0.51)	< 0.001	0.40 (0.30; 0.49)	< 0.001
Treatment Interventions	0.47 (0.37; 0.55)	< 0.001	0.39 (0.29; 0.49)	< 0.001

*r* Spearman correlation coefficient, *95% CI* 95% Confidence interval, p refers to Spearman correlation test.

IMPACT-III and PedsQL scores showed a good correlation (r = 0.75, 95% CI 0.69–0.80). All the IMPACT-III domains exhibited positive correlation coefficients with PedsQL domains (Table 3). Physical Health Summary Score domain on PedsQL was strongly correlated with Bowel Symptoms domain (r = 0.64) and with the Systemic Symptoms domain (r = 0.67) on IMPACT III. Psychosocial Health Summary Score was moderately correlated to all IMPACT-III domains, ranging from 0.42 to 0.63.

#### Discriminant validity and reproducibility

**Figure 1:**
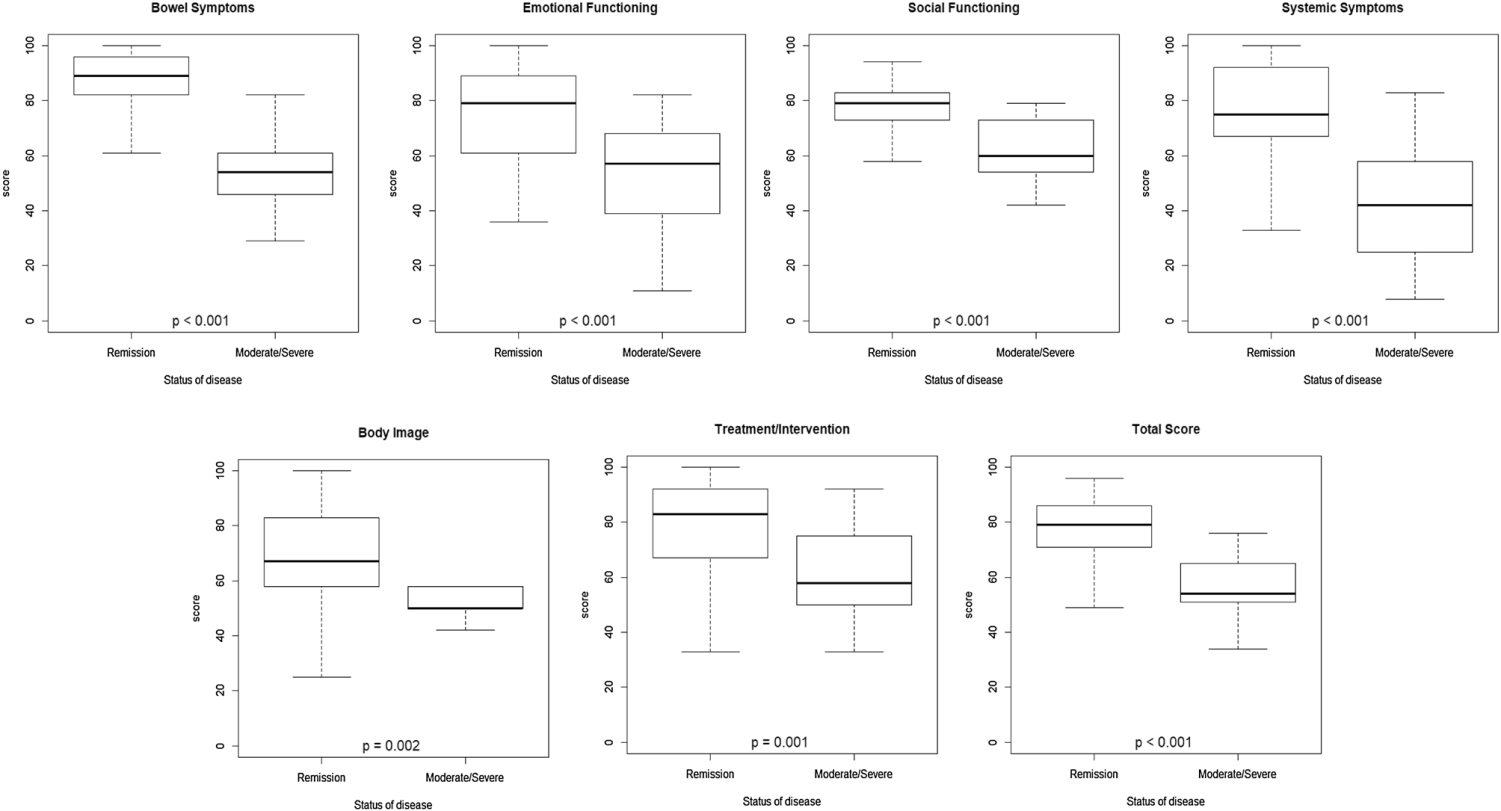
Discriminant validity for IMPACT-III questionnaire.

Discriminant validity performed using PUCAI/wPCDAI indices showed significant differences in all the IMPACT-III domains (Fig. 1). IMPACT-III questionnaire discriminated the severity of disease’s activity, i.e. patients with moderate-severe activity had significantly lower QoL scores in all IMPACT-III domains compared to patients in remission.

Reliability analysis was based on 176 subjects that compiled the questionnaire twice and had a stable disease. The 95% CI of the medians of the differences in IMPACT III scores between the two measurements always included zero for each domain, indicating no significant difference between the two time points (Supplementary Fig. S1). The coefficients were greater than 0.5 in all the domains highlighting a good level of reproducibility (Supplementary Table S2).

#### IMPACT-III questionnaire analysis

A median total IMPACT-III score of 76 (IQR 67–83) was observed, with no difference between CD and UC children (median scores: 78, IQR 68–84 and 76, IQR 66–82, respectively). Table 4 shows the comparison of IMPACT-III domain scores according to the type of disease. Bowel symptoms domain score was significantly higher in subjects affected by CD, while no significant difference between the two groups was found in the other domains.

**Table 4:** Health-related quality of life according to type of disease.

IMPACT-III domains	CD (n = 155)		UC (n = 123)		p
	Min; max	Median (IQR)	Min; max	Median (IQR)	
Bowel symptoms	32; 100	89 (79; 96)	21; 89	82 (68; 89)	0.001
Emotional functioning	14; 100	75 (64; 86)	7; 100	71 (56; 89)	0.158
Social functioning	44; 94	77 (71; 82)	42; 92	75 (71; 81)	0.31
Systemic simptoms	17; 100	75 (67; 83)	8; 100	75 (58; 83)	0.168
Body image	17; 100	67 (58; 83)	33; 100	67 (58; 83)	0.196
Treatment interventions	17; 100	75 (67; 92)	17; 100	75 (67; 83)	0.347
Total score	48; 95	78 (68; 84)	34; 96	76 (66; 82)	0.119

The total and single domain scores range from 0 to100, with 0 indicating the lowest HRQoL and 100 indicating the highest HRQoL. p-value refers to Wilcoxon rank sum test. *IQR* interquartile range, *min* minimum score value, *max* maximum score value.

#### Quantile regression analysis

Table 5 shows the results of the multiple quantile regression analysis at median level for each of the IMPACT-III domains. Female gender, lower BMI z-scores and relapses during the previous year were negatively associated to the Bowel Symptoms domain score. Remission (according to PGA) and CD diagnosis were associated with better QoL related to bowel symptoms (subjects with CD with a median score higher of 5 points compared to patients with UC).

**Table 5:** Factors associated with IMPACT-III domains.

Variables	Bowel symptoms		Emotional functioning		Social functioning		Systemic symptoms		Body image		Treatment/intervention	
	b	95% CI	b	95% CI	b	95% CI	b	95% CI	b	95% CI	b	95% CI
Gender (F vs M)	− 5.8	(− 8.3; − 2.4)	− 12.5	(− 16.3; − 3.7)	− 0.8	(− 4.4; 2.6)	− 11.5	(− 16; − 2.5)	− 8.3	(− 14.2; 1.6)	− 6.9	(− 13.7; − 1.0)
Age	− 0.0	(− 0.6; 0.5)	− 0.1	(− 1.2; 1.2)	− 0.2	(− 0.7; 0.4)	− 2.0	(− 3.2; − 1.1)	− 2.7	(− 3.7; − 1.1)	− 0.1	(− 1.2; 1.9)
Disease duration	− 0.2	(− 0.6; 0.3)	− 0.1	(− 1.6; 0.3)	− 0.5	(− 1.1; − 0.1)	− 0.9	(− 1.2; 0.4)	− 1.1	(− 2.1; 0.4)	− 1.0	(− 1.5; − 0.2)
Z-score BMI	1.5	(0.1; 2.8)	1.5	(− 3.2; 4.5)	− 0.6	(− 1.8; 1.5)	0.0	(− 2.4; 1.5)	− 0.3	(− 4.8; 1.3)	1.4	(− 2.6; 2.3)
PGA (active disease vs Remission)	− 10.2	(− 19.3; − 5.5)	− 20.6	(− 22.6; 1.8)	− 4.0	(− 5.9; 3.5)	− 8.0	(− 22.3; 17.1)	− 6.8	(− 21.3; 3.1)	− 22.7	(− 28.3; − 0.3)
PUCAI/w-PCDAI (Active disease vs Remission)	− 8.6	(− 10.6; 3.5)	10.6	(− 0.6; 14.4)	0.2	(− 7.0; 4.7)	− 7.7	(− 30.9; 8.7)	− 3.2	(− 17.0; 12)	11.7	(− 7.0; 16.0)
Type of disease (CD vs UC)	5.4	(1.9; 9.3)	2.9	(− 5.7; 7.3)	3.1	(− 1.9; 5.7)	4.8	(− 0.8; 15.4)	− 8.4	(− 15.2; 2.1)	− 1.9	(− 6.5; 5.7)
Relapses (yes vs no)	− 3.7	(− 10.1; − 0.1)	− 9.9	(− 22.1; − 6.1)	− 0.3	(− 5.8; 2.3)	− 5.4	(− 15.5; − 0.3)	4.3	(− 5.0; 11)	− 7.4	(− 16.5; 0.3)
Surgery (yes vs no)	0.6	(− 2.3; 2.9)	3.7	(− 2.2; 5.0)	− 1.3	(− 3.3; 2.3)	− 4.6	(− 6.3; 2.6)	5.2	(− 0.9; 10.6)	4.6	(− 1.5; 8.8)

Results of quantile regression analysis.

*b* quantile regression coefficient estimate, *95%CI* 95% confidence interval. Significant coefficients are marked in bold.

Both gender (females reporting 12 points less than males) and relapses had negative significant association with the Emotional Functioning domain.

Social Functioning domain was significantly associated with disease duration, a longer history of disease being associated to a worse social functioning.

Female gender, older age, longer disease duration and UC diagnosis were negatively associated with the QoL related to systemic symptoms.

Body Image score significantly decreased with increasing age (in median of three point for each year of age).

Treatment/Intervention score was significantly lower in females than males. The domain score significantly decreased with longer disease duration and in presence of active disease, as evaluated by clinicians (PGA).

**Figure 2:**
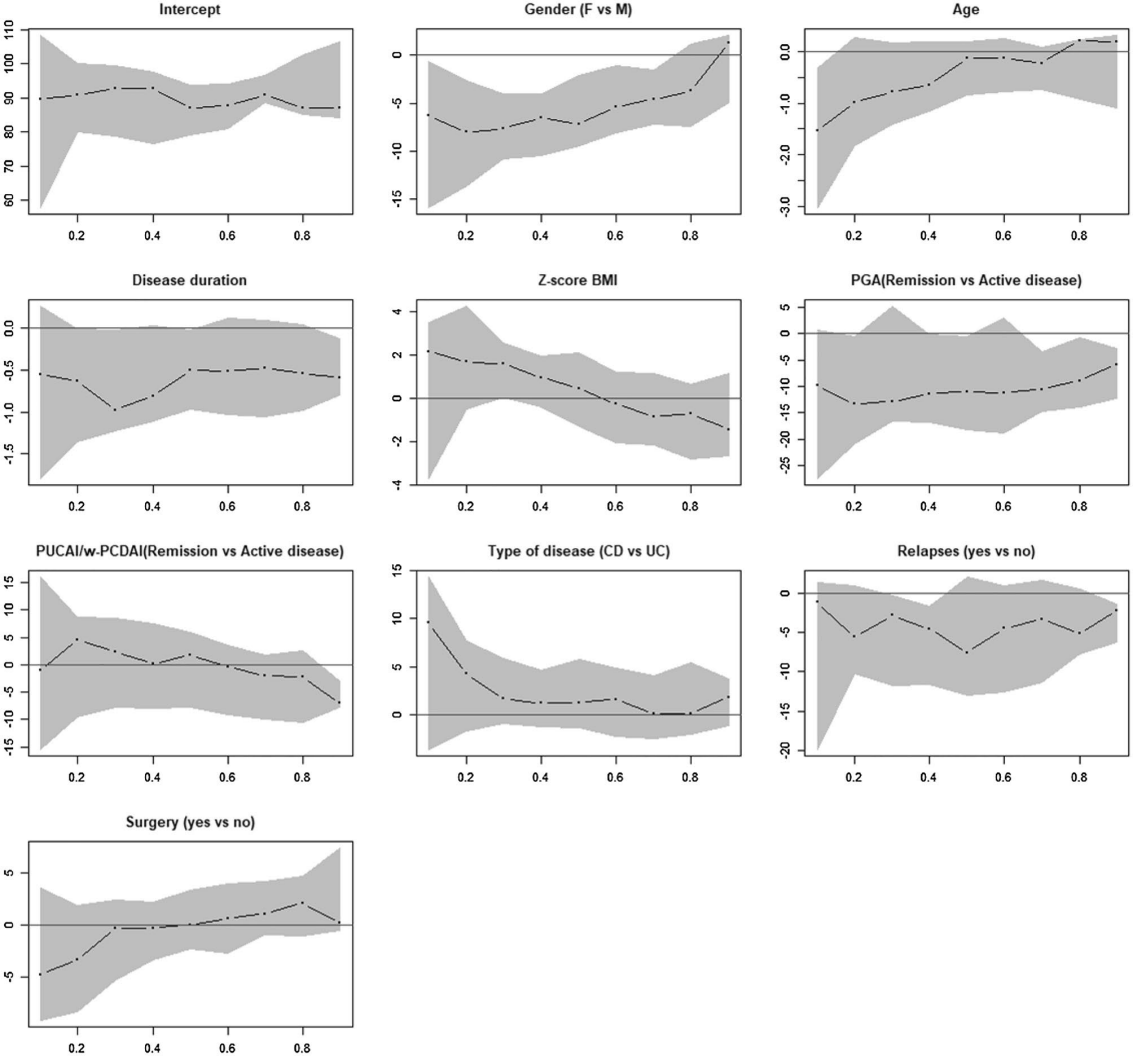
Effects of different variables on the IMPACT-III total score. The x-axis indicates the 9 deciles of the response distribution (score) and y-axis exhibits the effect of each variable (regression coefficients) and 95% confidence bands (grey area). If the confidence interval includes 0, the estimates cannot be considered statistically different from 0.

Figure 2 shows the effect of each variable on the IMPACT-III total score. Gender had a significant negative effect in the first 7 deciles of the distribution, while age was significantly associated with the total score in the first decile (subjects reporting the lowest QoL). Active disease, measured by PGA index, decreased the quality of life in the last three deciles of the score distribution (subjects with the highest QoL).

## Discussion

QoL is an important multidimensional issue in IBD care that needs to be addressed by the healthcare professionals involved in management of IBD. The knowledge of specific factors affecting or associated with QoL in a given cultural setting allows the prompt recognition of patients at risk for a low QoL and finally helps to improve the quality of care. This is the first multicenter study to assess factors associated with the HRQoL in a large cohort of Italian children and adolescents with IBD.

One of the major findings of our study is the negative association of female gender with most of the domains scores (Bowel Symptoms, Emotional Functioning, Systemic Symptoms and Treatment/Intervention) and with the total IMPACT-III score. While a similar effect has been well described in adult studies^19–21^, pediatric data are less consistent^22,23^. Previous studies reported a lower score in the “body image domain” for females which might be correlated with impaired QOL, a finding that is not confirmed by our study. It is possible that fears of body change and attractiveness is much more relevant in women compared to young females, while possible explanations of the reduced QoL in younger girls include a different perception of illness compared to boys, a greater concern on having symptoms or relapse or on taking long term medications. Furthermore, in comparison to men, women with IBD are more exposed to the risk of psychological disturbances (particularly anxiety)^24–26^ and social difficulties (e.g. in work productivity)^27,28^ and this can at least partially explain their reduced QoL as adults.

Another vulnerable category of patients are adolescents, as shown by the lower QoL reported by teens compared to younger children. Specifically, age was associated with a reduced scores in the Systemic Symptoms (2 points for each year) and the Body Image domains (2.7 points for each year), in line with previous studies^22,29^. The latter finding can be associated to a different perception and fear of body changes (related to cosmetic effects of drugs or to the diseases itself) in adolescents, with a reflection on QoL. Older children can sometimes deny or under report symptoms to minimize their disease and this can lead to an under estimation of disease activity (based on clinical scores) and to a delay in treatment intensification, with a subsequent disease flare. Both the twisted coping strategy and the worsening symptoms can ultimately affect their IBD related QoL. However, we found the effect of age on the total QoL score limited to subjects with the lowest QoL (first decile). This suggest that it is a specific subgroup of adolescents, where other factors contribute, to be particularly vulnerable to a reduced QoL. Our data warn about an early impairment of the QoL of girls and teenagers with IBD compared to boys, independent from other factors (such as social or work factors), and suggest the need of a careful monitoring of the QoL especially in female patients.

Our large multicenter experience does not support a significant association between the duration of disease and the total IMPACT-III score, therefore it seems appropriate to attribute the impaired QoL of older children to the vulnerable phase of development they are facing, rather than to the effect of the longer disease duration. It’s well described also in other chronic conditions (e.g. celiac disease)^30^ that accepting and coping with a chronic and social limiting disease can be particularly challenging for adolescents, with a negative impact on the QoL. However, the interpretation of these findings should also consider the cross-sectional design of our study that did not allow a longitudinal evaluation of possible temporal changes in QoL.

We did not find any significant difference in the QoL between CD and UC patients, except in the Bowel Symptoms domain score, which was significantly higher in CD subjects. This is at variance with reports in adults, that generally indicate a lower QoL in patients with CD compared to UC^31^. Pediatric data from Swiss, Asian and Greek cohorts do not confirm this finding^29,32,33^, e.g. the only difference in the Greek study was a higher emotional QoL in subjects with CD compared to UC. Inconsistencies between pediatric and adult data could be associated with the age-related increase of disease-complications and surgery (generally more frequent in patients with CD). It is also important to notice that in our study children with UC experienced a higher number of relapses in the previous year, perhaps correlating with their worse HRQoL on the Bowel Symptoms. Furthermore females were less represented in our CD group and, considering the strong association between female gender and a worse QoL, this finding should at least partially explains the comparable QoL recorded in all the other domains between UC and CD patients.

Finally, our data confirm that disease activity significantly influences the patient-reported QoL, as shown by several findings: (a) the univariate analysis shows that patients in remission reported higher total and subdomains scores, (b) in the quantile analysis, remission was associated to better QoL related to bowel symptoms and treatment/interventions and to a higher total QoL score. Furthermore, experience of relapses in the previous year significantly affected the Bowel Symptoms, the Emotional Functioning and the Systemic Symptoms QoL. Several previous studies indicated disease activity as a major factor associated with low QoL both in children^6,8^ and in adults; adult studies have shown the negative effect of disease activity on QoL, irrespective of the type of questionnaire (generic or disease specific)^31^. Controlling disease activity is one of the main objectives in treating IBD patients and this leads not only to remission of symptoms and achievement of mucosal healing, but also to improvement of psycho-social aspects and eventually of health-related QoL. Nevertheless it is important to notice how, in our experience, some domain scores were not associated to measures of disease activity (e.g. Emotional and Social Functioning), indicating that some aspects of QoL need to be specifically investigated and addressed by physician, regardless the control of symptoms and disease activity. Administration of a questionnaire investigating QoL and collection of the results prior to a hospital visit could be a strategy to focus on specific aspects of QoL, frequently underinvestigated during a medical interview, and to support the patient with different health professionals, such as a psychologist or a social assistant.

In line with previous pediatric studies^7–10^, our results confirm the good internal consistency of all the different domains of the IMPACT-III questionnaire and an appropriate concurrent validity (as shown by a comparison with the generic PEDsQL questionnaire). Furthermore, the test demonstrated good reproducibility and high discriminant validity, as shown by the capacity of separating patients according to disease’s activity (Fig. 1). Lastly, the questionnaire was easy to understand and appropriately completed by children and adolescents, as demonstrated by the low number of missing responses. Our findings definitively demonstrate, in a large multicenter setting, that the Italian version of the IMPACT-III questionnaire is a reliable instrument to assess QoL in IBD pediatric patients, supporting the use of this tool for clinical and research purposes.

Strengths of our study are the multi-center involvement and the large size of the sample, compared to previous reports. We were able to test the validity of the questionnaire with different approaches, including the test–retest reliability, by using an adequate period of reassessment. We are also aware that the cross-sectional design of our study limits the identification of longitudinal factors impacting QoL, nevertheless it allows to estimate factors associated with QoL.

## Conclusion

This study confirms the validity of the IMPACT III questionnaire in assessing QoL in a large sample of Italian children with IBD. We identified some characteristics potentially associated to QoL, suggesting the need of a special attention to a more vulnerable subgroup of IBD children (females, older children, UC subgroup and particularly patients with active disease and growth impairment).

## Supplementary Information

Supplementary Information.
