# Supplementary material for: Factors associated with quality of life in Italian children and adolescents with IBD
Source: Sci Rep. 2021 Sep 10;11:18076. doi: 10.1038/s41598-021-97661-1 (PMC8433211; doi:10.1038/s41598-021-97661-1)
Supplement: Supplementary file 1 — Supplementary Information. [file 41598_2021_97661_MOESM1_ESM.docx]

**Title: Factors associated with quality of life in Italian children and adolescents with IBD**

**Authors: Simona Gatti PhD^1^, Giada Del Baldo MD^1^, Giulia Catassi MD^2^, Andrea Faragalli PhD^3^, Marina Aloi PhD^2^, Matteo Bramuzzo MD^4^, Giulia D’Arcangelo MD^3^, Enrico Felici MD^5^, Maurizio Fuoti MD^6^, Sara Lega MD^4^, Roberto Panceri MD^7^, Maria Pastore MD^8^, Francesca Penagini PhD^9^, Rosaria Gesuita PhD^3^, Carlo Catassi MD^1^**

1. **Department of Pediatrics, Polytechnic University of Marche, G. Salesi Children's Hospital, via Corridoni 11, 60123, Ancona (Italy);**
2. **Department of Maternal and Child Health, Pediatric Gastroenterology and Liver Unit, Umberto I Hospital, Sapienza University of Rome, viale del Policlinico 105, 00161 Rome (Italy)**
3. **Centre of Epidemiology and Biostatistics, Università Politecnica delle Marche, via Tronto 10/a, 60126 Ancona, (Italy);**
4. **Gastroenterology, Digestive Endoscopy and Nutrition Unit, Institute of Child and Maternal Health, IRCCS “Burlo Garofolo”, via dell’Istria 65, 34137 Trieste (Italy)**
5. **5. Pediatrics and Pediatric Emergency Unit “U.Bosio” center for Pediatric Digestive Diseases, The Children Hospital, Azienda Ospedaliera SS. Antonio e Biagio e Cesare Arrigo, Spalto Marengo 46, 15121Alessandria (Italy)**
6. **Gastroenterology and GI endoscopy, University Department of Pediatrics, Children’s Hospital, Piazzale Spedali Civili 1, 25123Brescia (Italy)**
7. **Clinica Pediatrica, Università Milano Bicocca, Fondazione MBBM, Ospedale San Gerardo, Via Cadore, 20900 Monza (Italy)**
8. **IRCCS Casa Sollievo della Sofferenza-Pediatria, Viale Padre Pio, 7d, 71013 San Giovanni Rotondo (Italy)**
9. **Clinica Pediatrica, Ospedale dei Bambini “V. Buzzi”, Università degli Studi di Milano, Via Lodovico Castelvetro 32, 20154 Milano (Italy)**

**Authors e-mail: Simona Gatti: simona.gatti@hotmail.it; Giada Del Baldo: giadadelbaldo@gmail.com; Giulia Catassi: giulia.catassi@gmail.com; Andrea Faragalli: a.faragalli@staff.univpm.it; Marina Aloi: marina.aloi@uniroma1.it; Matteo Bramuzzo: matteo.bramuzzo@burlo.trieste.it; Giulia D’Arcangelo: giuliadarcangelo87@gmail.com; Enrico Felici: enrico.felici@ospedale.al.it; Maurizio Fuoti: maurysgm@yahoo.it; Sara Lega: sara.lega@burlo.trieste.it; Roberto Panceri: panceri-37392@asst-monza.it; Maria Pastore: mariapastore1@virgilio.it; Francesca Penagini: francesca.penagini@asst-fbf-sacco.it; Rosaria Gesuita: r.gesuita@staff.univpm.it; Carlo Catassi: c.catassi@univpm.it**

**Address for correspondence:**

**Dr. Simona Gatti, Department of Pediatrics, G. Salesi Children’s Hospital, Azienda Ospedaliero Universitaria Ospedali Riuniti, Ancona, via Corridoni 11 60123, Ancona.**

**Phone: +390715962114 ; Fax: +3907136284; e-mail: simona.gatti@hotmail.it**

**Funding:** No funding.

**Supplementary table S1**

**IMPACT-III domains' score according to participating centres**

|  | **Ancona** | **Brescia** | **Milano** | **Pesaro** | **Roma** | **San Giovanni Rotondo** | **Trieste** | **Monza** | **p** |
| --- | --- | --- | --- | --- | --- | --- | --- | --- | --- |
| **IMPACT III Domains** | n=32 | n=24 | n=36 | n=6 | n=51 | n=42 | n=77 | n=10 |  |
| **Bowel symptoms** | 84 (79-89) | 89 (75-96) | 89 (79-96) | 81 (73-96) | 86 (70-93) | 79 (65-86) | 89 (79-96) | 82 (74-88) | 0.021* |
| **Emotional functioning** | 73 (60-86) | 82 (67-93) | 73 (60-89) | 88 (82-93) | 75 (59-84) | 66 (54-82) | 79 (61-89) | 72 (65-79) | 0.084 |
| **Social functioning** | 77 (71-81) | 78 (68-83) | 75 (70-84) | 77 (76-83) | 77 (71-83) | 75 (71-79) | 77 (73-83) | 76 (69-81) | 0.749 |
| **Systemic simptoms** | 75 (58-92) | 67 (48-77) | 75 (67-85) | 79 (69-96) | 75 (63-83) | 75 (67-92) | 75 (58-83) | 58 (37-73) | 0.075 |
| **Body imgae** | 67 (50-75) | 75 (50-83) | 67 (58-75) | 75 (62-94) | 67 (58-83) | 71 (58-83) | 67 (50-83) | 58 (50-73) | 0.563 |
| **Treatment Interventions** | 75 (67-92) | 83 (65-92) | 79 (67-92) | 83 (71-96) | 75 (58-83) | 71 (58-90) | 75 (58-83) | 83 (77-90) | 0.526 |
| **Total score** | 76 (68-82) | 80 (66-87) | 76 (70-85) | 79 (74-91) | 77 (69-81) | 72 (65-82) | 78 (67-85) | 70 (68-80) | 0.472 |

p-values refer to Kruskal-Wallis test; *Significant difference between San Giovanni Rotondo and Trieste

**Supplementary Figure S1**

**Distribution of the differences in the IMPACT III score between the two measurements of the test-retest.**

**
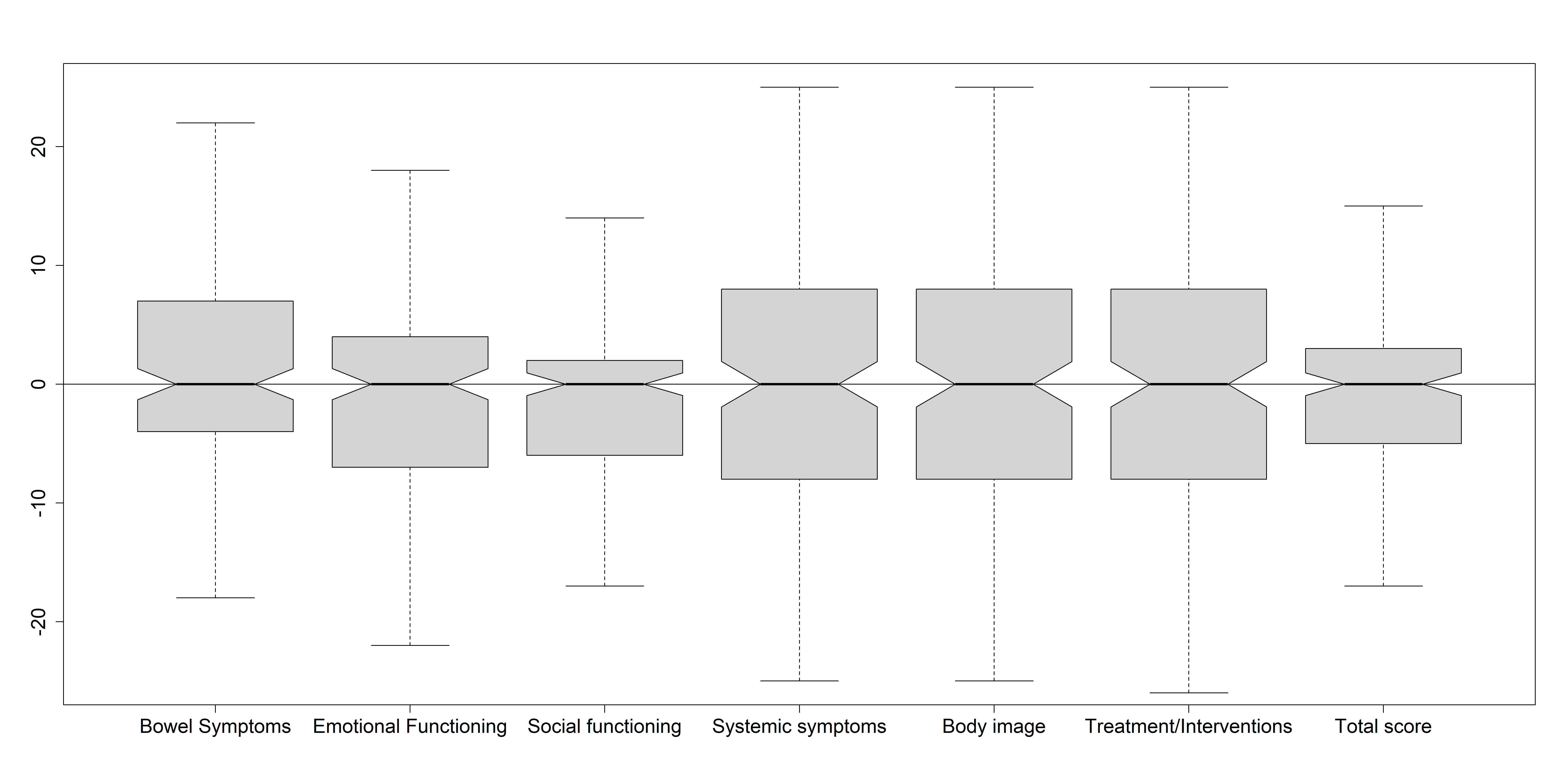
**

**Supplementary Table S2**

**Test-retest analysis results**

| IMPACT III Domains | Median score at 1^st^ time point (95%CI) | Median score at 2^nd^ time point (95%CI) | Median of differences in score between the two time points (95% CI) | CCC (95% CI) |
| --- | --- | --- | --- | --- |
|  |  |  |  |  |
| Bowel symptoms | 86 (73; 93) | 86 (71; 93) | 0 (-4; 7) | 0.64 (0.54; 0.72) |
| Emotional functioning | 75 (57; 89) | 75 (57; 89) | 0 (-7; 4) | 0.66 (0.57; 0.74) |
| Social functioning | 77 (71; 82) | 79 (72; 83) | 0 (-6; 2) | 0.56 (0.45; 0.66) |
| Systemic symptoms | 75 (58; 92) | 57 (58; 83) | 0 (-8; 8) | 0.58 (0.47; 0.67) |
| Body image | 67 (58;83) | 67 (58; 83) | 0 (-8; 8) | 0.73 (0.65; 0.79) |
| Treatment Interventions | 75 (67; 92) | 75 (67; 92) | 0 (-8; 8) | 0.6 (0.50; 0.69) |
| Total score | 76 (66; 84) | 77 (67; 84.5) | 0 (-5; 3) | 0.66 (0.57; 0.74) |
